# Supplementary material for: A Global Screen for Assembly State Changes of the Mitotic Proteome by SEC-SWATH-MS
Source: Cell Syst. 2020 Feb 26;10(2):133–155.e6. doi: 10.1016/j.cels.2020.01.001 (PMC7042714; doi:10.1016/j.cels.2020.01.001)

Q5TFE4 | NT5D1_HUMAN | NT5DC1 NT5C2L1 LP2642
Monomer MW [kDa]: 51.845 Monomer expected elution fraction: 46

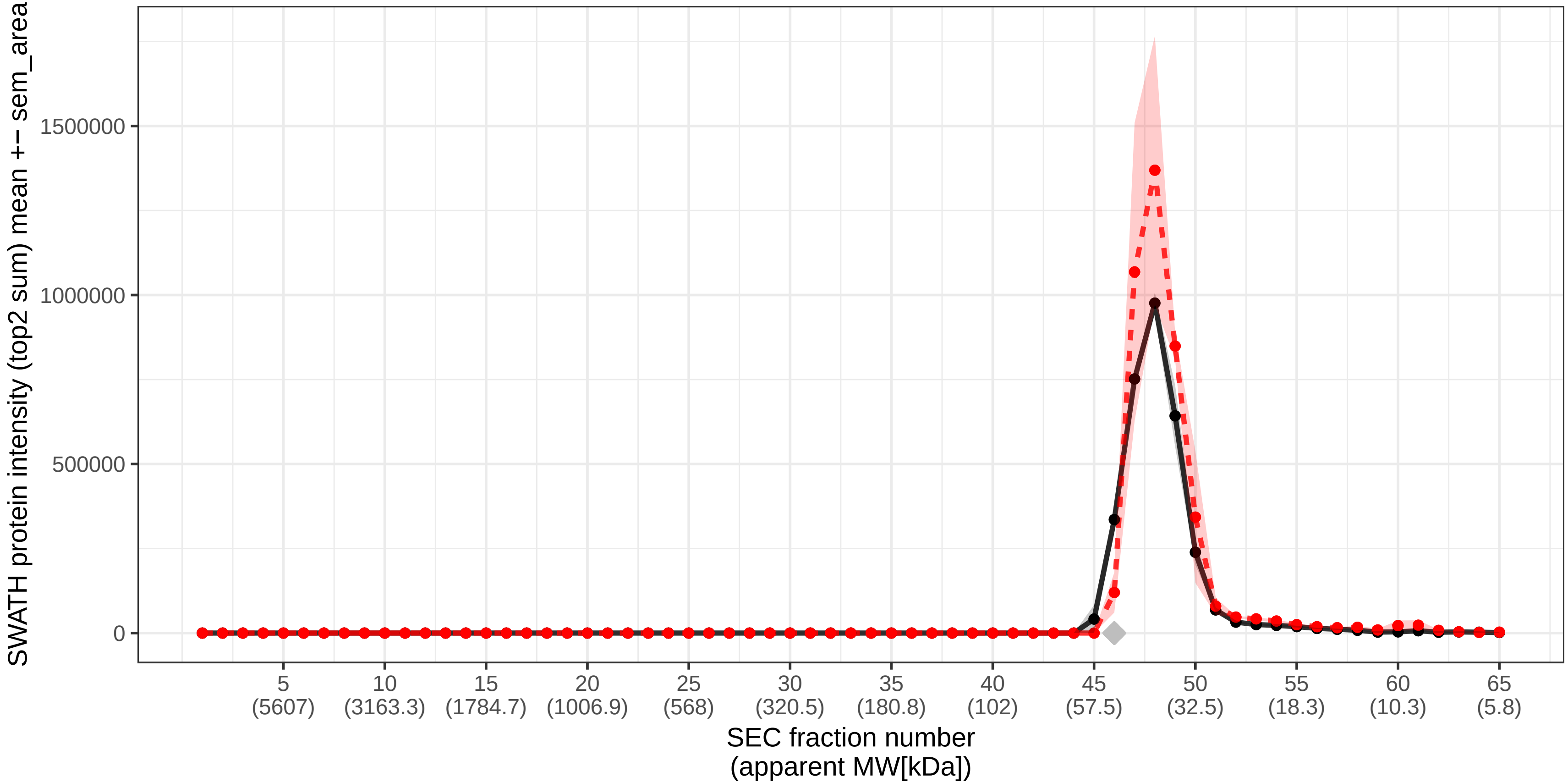

Supplement: Data S1. SEC-SWATH-MS Protein Chromatograms, Related to Figure 1 [file mmc6.zip › SECchrom_Q5TFE4_NT5D1_HUMAN_NT5DC1_NT5C2L1_LP2642.pdf]
